# The relationship of the musculocutaneous nerve to the brachial plexus evaluated by MRI

**DOI:** 10.1007/s10877-015-9807-3

**Published:** 2015-11-19

**Authors:** Trygve Kjelstrup, Axel R. Sauter, Per K. Hol

**Affiliations:** 1 Department of Anaesthesiology, Diakonhjemmet Hospital, Pb 23 Vinderen, 0319 Oslo, Norway; 2 The Intervention Centre, Oslo University Hospital, Rikshospitalet, Oslo, Norway; 3 Division of Emergencies and Critical Care, Department of Anaesthesiology, Oslo University Hospital, Rikshospitalet, Oslo, Norway; 4 Institute of Clinical Medicine, University of Oslo, Oslo, Norway

**Keywords:** Brachial plexus, Musculocutaneous nerve, MRI scan

## Abstract

Axillary plexus blocks (AXB) are widely used for upper limb operations. It is recommend that AXB should be performed using a multiple injection technique. Information about the course and position of the musculocutaneous nerve (MCN) is of relevance for AXB performance. The objective of this study was to examine the position of the MCN and its relationship to the axillary sheath using MRI. 54 patients underwent an AXB with 40 ml of local anaesthetic before MRI examination. The course of the MCN and the position where it left the axillary sheath and perforated the coracobrachial muscle (MCN exit point), in relation to the axillary artery and the block needle insertion point in the axillary fold, were recorded. The MCN was seen clearly in 23, partly in 26, and not identified in five patients at the MCN exit point. The mean distance from the insertion point of the block needle in the axillary fold to the MCN exit point was 36.8 mm (SD = 18.9, range: 0–90.5). In 37 patients the MCN exit point was positioned inside the Q_1_ quadrant (lateral anterior to the axillary artery) and in 11 patients inside the Q_2_ quadrant (medial anterior to the axillary artery). There is a wide variability as to where the musculocutaneous nerve (MCN) leaves the axillary sheath. Therefore multiple injection techniques, or the use of a proximally directed catheter, should be appropriate to block the MCN.

## Introduction

Axillary plexus blocks (AXB) are widely used as an anaesthetic method for upper limb operations. It is recommend that AXB should be performed using a multiple injection technique [1]. At terminal nerve level, the musculocutaneous nerve (MCN) is usually positioned outside the axillary sheath. There is an ongoing discussion as to whether a selective block of the MCN is necessary to achieve a successful AXB [2, 3]. The objective in this study was to examine the position of the MCN and its relationship to the axillary sheath using MRI. Information about the course of the MCN can indicate how, and where, the MCN can be blocked when performing AXB.

## Methods

Ethical approval for this study (Ethical Committee No S-04115) was provided by the Committee for Medical Research Ethics, Region South East (REK Sør-Øst), Pb 1130 Blindern, 0318 Oslo, Norway on June 8th, 2004 as for a previous study [4].

The study was conducted at Oslo University Hospital, Rikshospitalet, in the period November 10th, 2014–February 1st, 2015. Forty five patients from a study on axillary brachial plexus blocks [5] and nine patients from a pilot study [4] were analysed as one group using the existing MRI data.

All patients underwent an AXB with 40 ml of local anaesthetic (LA) injected before MRI scanning (Achieva 3T, Philips Healthcare, The Netherlands) was performed [4, 5]. MRI scanning was performed immediately after completion of the block-procedures.

The patients were examined in the horizontal supine position with the arms adducted. T1- and T2-weighted images were obtained according with a previously described protocol [4], and the images underwent a consensus assessment by the three authors.

**Fig. 1:**
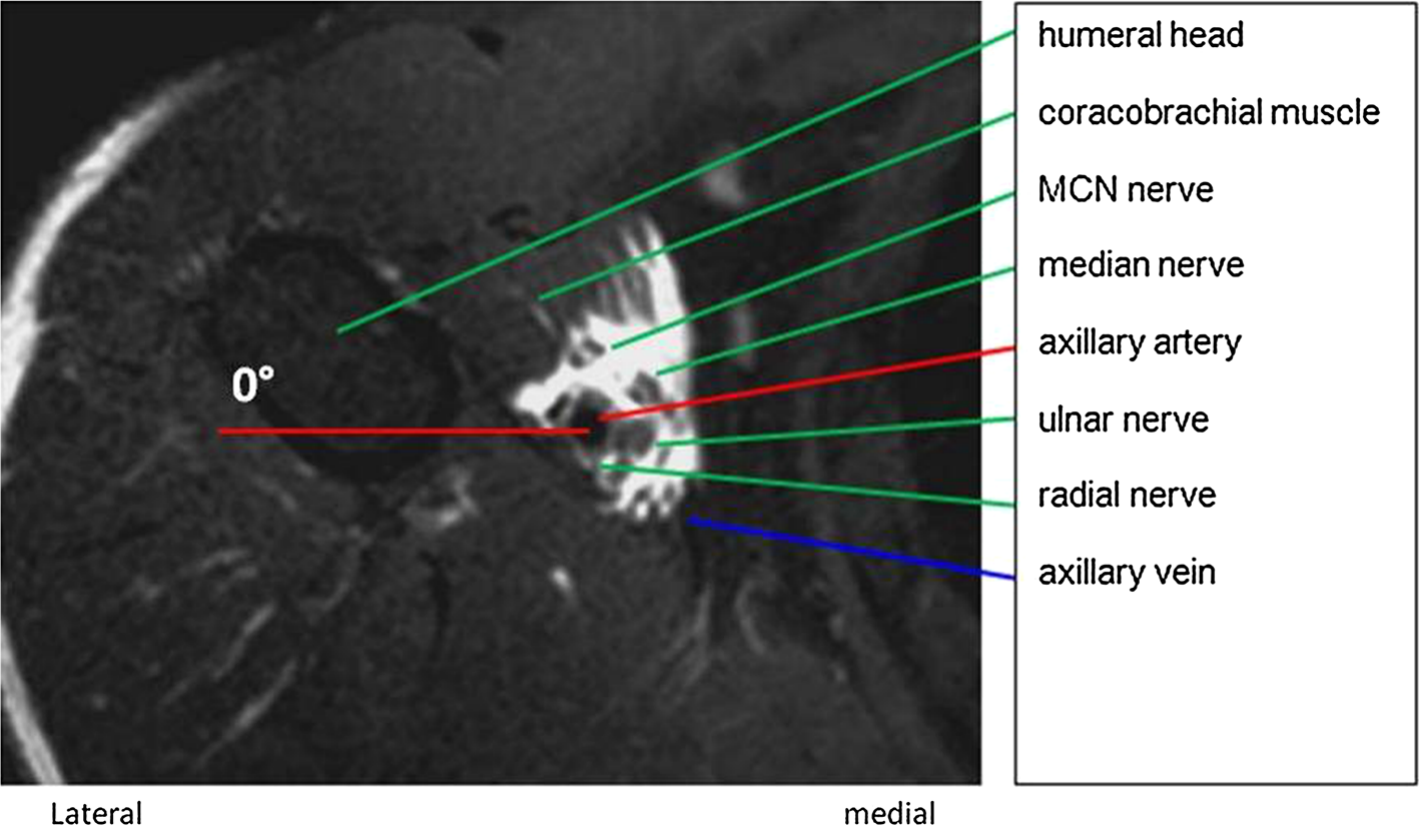
MRI of the right axilla, cross-sectional view. T2-weighted image with fat suppression from a patient with a successful sensory block after a single local anaesthetic (LA) injection. The LA appears white. The musculocutaneous nerve (MCN) is clearly seen before entering the coracobrachial muscle. The lateral horizontal line (*red*) towards the humerus head was defined as the zero angel line

**Fig. 2:**
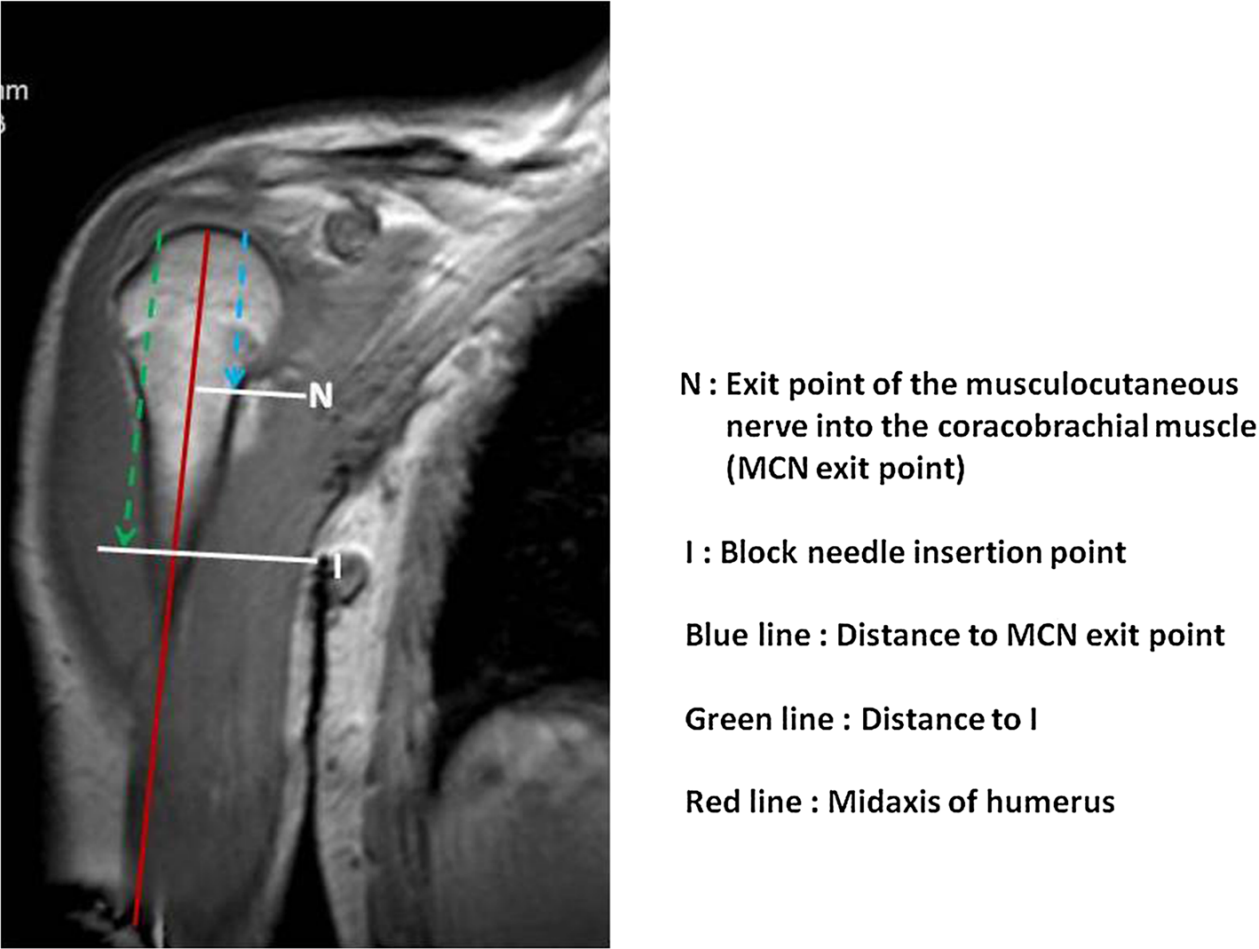
MRI of right shoulder, coronal view, in one of the patients. T1-weighted image. With a reference point at the top of the humeral head, the distances (*blue* *line*, *green line*) to the MCN exit point (*N*) and the insertion point of the block needle (*I*) were measured. The humeral shaft (*red line*) served as reference line

The course of the MCN and the surrounding anatomy was analysed in the short axis view (cross-sectional, Fig. 1) and in the long axis view (coronal, Fig. 2). The MCN exit point was defined as the position where the nerve left the neurovascular bundle into the coracobrachial muscle (Fig. 1).

The MCN exit point was measured with reference to the top of the humeral head (Fig. 2). Similarly, the most cranial part of the axillary fold was defined as the block needle insertion point, and the distance from this point to the top of the humeral head was recorded (Fig. 2). The difference between these two measurements gave the distance from the block needle insertion point to the MCN exit point.

The visibility assessment was performed at the MCN exit point and was scored as 0 = not visible, 1 = partly visible and 2 = clear visible.

**Fig. 3:**
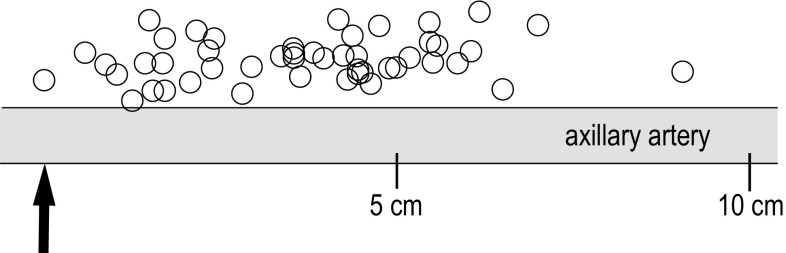
The exit points of the musculocutaneous nerve into the coracobrachial muscle in relation to the insertion point of the block needle (*arrow*) in the axillary fold, and in relation to the centre of the artery

**Fig. 4:**
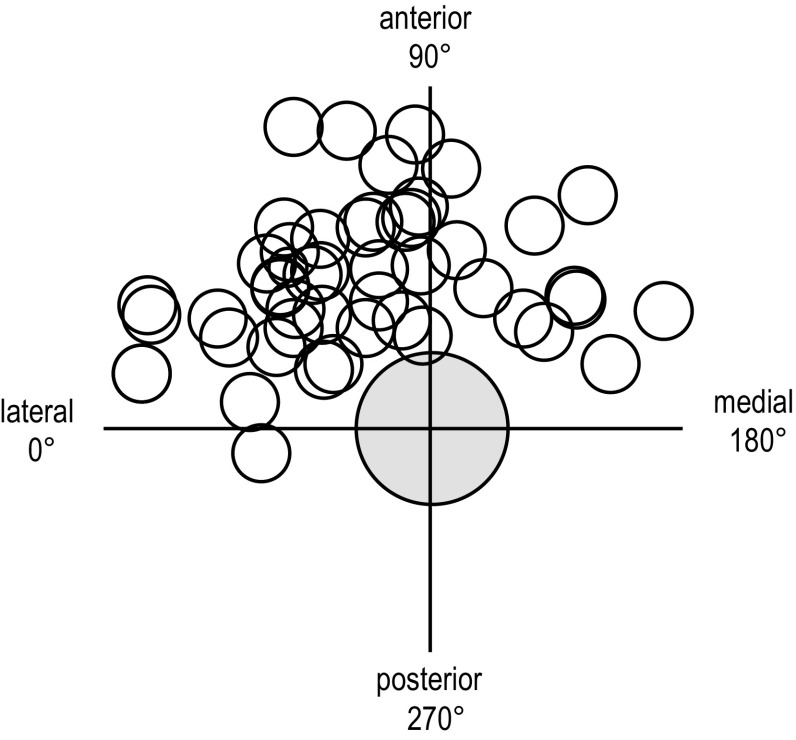
The exit points of the musculocutaneous nerve into the coracobrachial muscle in relation to the quadrants and the axillary artery (*centre*). The four quadrants: Q_1_ = the lateral anterior (0°–90°), Q_2_ = the medial anterior (90°–180°), Q_3_ = the medial posterior (180°–270°) and Q_4_ = the lateral posterior (270°–360°) quadrant

The positions of the MCN exit points were plotted graphically. The estimated diameters were 8 mm for the artery and 3 mm for the nerves (Figs. 3 and 4).

The MCN exit point was also described by the four quadrant positions: Q_1_ = the lateral anterior, Q_2_ = the medial anterior, Q_3_ = the medial posterior and Q_4_ = the lateral posterior quadrant (Fig. 4). The angel and distance from the mid axis of the axillary artery to the MCN exit point was recorded, and a lateral horizontal line towards the humerus was defined as the zero angel line (Fig. 1).

### Statistics

For statistical analysis, we used SPSS version 22 (IBM Corporation, Armonk, New York, USA). Data were described by mean, standard deviation (SD), range and counts.

## Results

Of the 54 patients, 30 had a plexus block on the right and 24 on the left side. In 23 patients the MCN exit point was clearly and 26 patients partly visible. In 5 patients the MCN exit point could not be identified.

The mean distance from the top of the humeral head to the insertion point of the block needle was 98.4 mm (SD = 10.9, range: 81.4–132). The MCN exit point was positioned at a mean distance of 61.5 mm (SD = 17.6, range: 29.6–106.7) from the humeral head. Thus, the mean distance from the insertion point of the block needle in the axillary fold to the MCN exit point was 36.8 mm (SD = 18.9, range: 0–90.5; Fig. 3).

The mean angel from the lateral horizontal line to the MCN exit point was 69.4º (SD = 39.7°, range: −8.1° to 160.2°) for all patients (Fig. 4). In 37 patients the MCN exit point was positioned inside the Q_1_ quadrant (lateral anterior to the axillary artery) and in 11 patients inside the Q_2_ quadrant (medial anterior to the axillary artery). In only one patient the MCN exit point was found in the Q_4_ quadrant at −8.1° to the zero angel line (Fig. 4). The average distance from the centre of the artery to the MCN exit point was 10.8 mm (SD = 3.0, range: 4.9–17.5).

## Discussion

The MRI of the axillary brachial plexus from 54 patients showed a wide variability as to where the musculocutaneous nerve (MCN) leaves the axillary sheath. The MCN exit points were mainly localized in the lateral anterior (Q_1_) and the medial anterior (Q_2_) quadrant and spread over a distance of approximately 10 cm proximally to the axillary fold.

The large distance between the MCN exit point and the insertion point of the block needle in the axillary fold, which we found in our study, can explain the incomplete success rates in some axillary blocks techniques when the MCN is not selectively blocked. Block success rates of the MCN can range from 27 to 40 % as seen in a study without a selective block of the nerve [6]. With a selective block on the other hand, success rates for MCN blocks above 90 % are achieved [6–8].

According to the concept of a continuous neurovascular sheath, a sufficiently high injection volume should give an adequate proximal spread to the MCN [9]. However, several studies have demonstrated that the brachial plexus sheath is divided by connective tissue septa into multiple compartments [10, 11]. LA spread, especially in the cross-sectional plane, can be inhibited. The wide spread of the MCN exit points in the two anterior quadrants favour a multiple injection technique, or a selective block, in order to achieve a successful AXB.

In our previous study, block success of the MCN was 100 % when an LA volume of 40 ml was applied with multiple injections through a proximal catheter combined with two deposits lateral and medial to the axillary artery (without blocking the MCN selectively). When only a proximal catheter was used for LA injection, the success rate for MCN block decreased to 73 % [5].

Non-ultrasound-guided and single injection axillary plexus block techniques have typically used high doses of LA [12–14]. We therefore considered the 40 ml LA volume used in our study as adequate in order to fill up the axillary sheath [15]. When nerves are detected individually by ultrasound guidance or electrical nerve stimulations, lower LA volumes are appropriate [12, 16, 17].

Our study has several limitations. MRI measurements are substantially observer dependant. Therefore the images were discussed among the three authors, and a consensus agreement was achieved. In five of the 54 patients (9 %) the MCN exit point could not be identified. One reason for our missing data may be that the MCN exit point was outside the scanning field. However, the MCN can sometimes be missing, or it demonstrates unusual anatomy with an alternative course, passing over the surface of the coracobrachial muscle instead of perforating it [18–20]. In the study of Guerri-Guttenberg et al. [19] the MCN was absent at a frequency of 3.6 %, and in 11 % of the dissections the nerve did not perforate the coracobrachial muscle.

The MRI examination was performed just after the LA had been injected. The distortion of the anatomy [4], due to the injection, may have altered the MRI measurements. Especially the distance between the MCN exit point and the artery could be increased.

Because of the closed scanner construction, our MRI examinations were performed with the arm adducted. Distances and nerve positions might differ when the arm is abducted, as in the typical position when performing the AXB.

## Conclusion

There is a wide variability as to where the musculocutaneous nerve (MCN) leaves the axillary sheath. Therefore multiple injection techniques, or the use of a proximally directed catheter, should be appropriate to block the MCN.
